# Heterogeneous nuclear ribonucleoprotein A/B: an emerging group of cancer biomarkers and therapeutic targets

**DOI:** 10.1038/s41420-022-01129-8

**Published:** 2022-07-25

**Authors:** Ya Lu, Xinyue Wang, Quan Gu, Juan Wang, Ying Sui, Jianzhong Wu, Jifeng Feng

**Affiliations:** Nanjing Medical University Affiliated Cancer Hospital & Jiangsu Cancer Hospital & Jiangsu Institute of Cancer Research, Nanjing, China

**Keywords:** Tumour biomarkers, Targeted therapies

## Abstract

Heterogeneous nuclear ribonucleoprotein A/B (hnRNPA/B) is one of the core members of the RNA binding protein (RBP) hnRNPs family, including four main subtypes, A0, A1, A2/B1 and A3, which share the similar structure and functions. With the advance in understanding the molecular biology of hnRNPA/B, it has been gradually revealed that hnRNPA/B plays a critical role in almost the entire steps of RNA life cycle and its aberrant expression and mutation have important effects on the occurrence and progression of various cancers. This review focuses on the clinical significance of hnRNPA/B in various cancers and systematically summarizes its biological function and molecular mechanisms.

## Facts

- Heterogeneous nuclear ribonucleoproteins (hnRNPs) are the most abundant nuclear protein in higher eukaryotes and a class of typical and acknowledged RNA binding proteins.
- HnRNPA/B subfamily is the core members of hnRNPs and closely associated with cancer initiation and progression.
- HnRNPA/B shows dynamic changes in human cancer progression and is identified as a promising biomarker of cancer.
- The regulatory network of hnRNPA/B in cancer is complex and diverse and has received widespread attention.
- Inhibitors targeting hnRNPA/B are continually being explored for clinical use, and a number of compounds of food, plant or traditional Chinese herbal are increasingly being found to contribute in cancer therapy by targeting hnRNPA/B.

## Open Questions

- What are the specific mechanisms that cause the signature alterations of hnRNPA/B in cancer?
- Whether there are distinguished functions among hnRNPA/B members or even different isoforms?
- Why do members of the same hnRNPA/B subfamily appear to have contrasting expression and effects in the same cancer?
- Can hnRNPA/B achieve true clinical translation as a marker for cancer surveillance and a target for cancer treatment?

## Introduction

Heterogeneous nuclear RNAs (hnRNAs) are the major transcripts of RNA polymerase II in eukaryotes. The nascent mature hnRNAs indiscriminately bind to numerous proteins to form complexes during transcription, and heterogeneous nuclear ribonucleoproteins (hnRNPs) are precisely the integral protein components of these complexes [1]. HnRNPs are the most abundant nuclear protein in higher eukaryotes and a class of typical and acknowledged RNA binding proteins (RBPs) [2]. About 20 major members (A-U) of the hnRNPs family were separated from eukaryotic cells [3]. Among them, hnRNPA1 and A2 alone have been identified to account for approximately 60% of the hnRNPs mass and are the important particles of hnRNPs’ biological roles in the life cycle [4].

The hnRNPA/B subfamily is the core member of hnRNPs, mainly including four isoforms hnRNPA0, A1, A2/B1, and A3 [5]. Recently, the biological value of hnRNPA/B has been widely discussed and highly valued. HnRNPA/B members share similar biogenesis and penetrate extensively and deeply into all levels of cellular RNA metabolism, participating in DNA binding, RNA splicing and trafficking, and mRNA translation and stability [6]. Although the structural characterization of hnRNPA/B and its role in RNA homeostasis have been studied and summarized in detail [7], the different biological functions of its four isoforms in human cancers are vital topics that cannot be overlooked. This review focuses on recent insights into the role and value of hnRNPA/B in cancer. This is the first comprehensive and systematic review of the differential expression, biological function, molecular mechanisms and clinical significance of hnRNPA/B in multiple cancers, providing a research summary and theoretical support for hnRNPA/B members as promising cancer biomarkers and therapeutic targets.

## Profiles of hnRNPA/B: structure and intracellular localization

The ability of hnRNPA/B to co-package RNA into an array of regular ribonucleosomes is inextricably linked to its structural features [8]. HnRNPs consist of RNA-binding domains (RBDs) and auxiliary domains [9], which guiding hnRNPs to interact with target genes or other proteins by recognizing specific nucleotide sequences in the open reading frame (ORF) or untranslated region (UTR) [10]. Currently, RNA recognition motif (RRM), Arg-Gly-Gly (RGG) box and K-Homology (KH) motif are the main RBDs that have been authenticated maturely [11]. Among them, RRM is the most common and highly conserved sequence, shared by most hnRNPs family members [12]. While the number and spacing of RGG repeats vary considerably in different members [11]. And KH motif only presents in hnRNPE1, E2 and K [13]. As for the auxiliary domain, it is a dispersed and unstructured region that includes glycine-, alanine- or proline-rich domains [2, 9], perhaps influencing the RNA-binding site of hnRNPs to some extent [14]. In addition, the nuclear localization signal (NLS) and nucleo-cytoplasmic shuttling (NS) domains are also important components of hnRNPs, and their integrity is essential for mediating hnRNP nuclear imports [15].

To sum up, hnRNPA/B is a subgroup of proteins sharing similar structures consisting of two RRM and a glycine-rich domain that further encompasses an RGG box, an M9-NLS and a core prion-like domain (PrLD) [6, 16]. HnRNPA/B is predominantly localized in the eukaryotic nucleus and can accompany RNA transcripts into the cytoplasm in cooperation with the nuclear pore complex (NPC) [17]. It is reported that the GTPase Ran-GTP/GDP concentration gradient is crucial for maintaining the intracellular localization of hnRNPA/B [18]. Remarkably, hnRNPA/B is distinguished from a confusingly named protein, hnRNPAB (also known as CBF-A). Although they both share the characteristic RBD structural domain [19], the conserved amino acids that make up the primary structure differ from each other, so they are divided into two distinct subgroups, A and D [20]. Studies suggested that the completely different evolutionary directions between hnRNPA/B and hnRNPAB might lead to very different biological functions, but this review will not dwell on this but will focus only on the progress of hnRNPA/B family members in cancer.

HnRNPA/B possesses similar and distinct biological functions from other hnRNPs family members. Precisely, even individual members of the same hnRNPA/B subfamily exhibit diverse effects on the cancer microenvironment [21]. Consequently, distinguishing different hnRNPA/B members and understanding their specific biological functions in cancers will be of great significance.

## The roles of hnRNPA/B in cancers: molecular mechanisms and clinical significance

### HnRNPA0

HnRNPA0 is an important partner in RNA processing that can be phosphorylated by MAPKAP-K2 at Ser84 and induced by lipopolysaccharide (LPS) to assist in post-transcriptional regulation of specific mRNAs under inflammatory stimulation [22]. Recently, the abnormality of hnRNPA0 has gradually proved to be firmly associated with cancer development. Studies have shown that hnRNPA0 is located within the commonly deleted segment of 5q31.2 in myeloid neoplasms (MNs) with a del(5q). It is highly expressed in hematopoietic stem cells (HSCs) common-myeloid progenitors (CMPs) and megakaryocyte-erythrocyte progenitors (MEPs) and suppressed as cells differentiate towards different hematopoietic lineages. Meanwhile, a decreased dose of hnRNPA0 in therapy-related myeloid neoplasms (t-MNs) patients may contribute to leukemogenesis. Hence, haploinsufficiency of hnRNPA0 was considered as one of the key initiating mutations in the pathogenesis of MNs with a del(5q) [23]. Interestingly, hnRNPA0 mutation has also been found to be related to increased cancer incidence in a large family cursed with strong familial susceptibility to cancers [24].

Moreover, hnRNPA0 was also regarded as a strong promoter for various cancers such as hereditary colorectal cancer (CRC) [25], metastatic clear cell renal cell carcinoma (ccRCC) [26] and endometrial cancer (EC) [27]. It was reported that cancer-specific phosphorylated hnRNPA0 facilitated chromosomal alignment in mitosis and promoted CRC cell progression through RAB3GAP1-ZWINT1 cascade. The deactivation or deletion of the phosphorylated site of hnRNPA0 (Ser84) could weaken the interaction between hnRNPA0 and RAB3GAP1, thereby inducing proteasomal degradation of ZWINT-1 activated by Rab3 and CRC cell apoptosis [28]. In addition, hnRNPA0 was deemed as a “successor” to p53 for checkpoint control. Like p53, hnRNPA0 was activated by a checkpoint kinase (MK2) and simultaneously controlled cell cycle checkpoints. But unlike p53, hnRNPA0 repaired DNA damage caused by chemotherapy and drove cisplatin resistance by the post-transcriptional stabilization of p27(Kip1) and Gadd45α mRNAs [29, 30]. However, the translation process and oncogenic effects of hnRNPA0 could be hindered by lncRNA miR205HG in esophageal carcinoma (ESCA), in which hnRNPA0 was highly expressed [31].

**Fig. 1:**
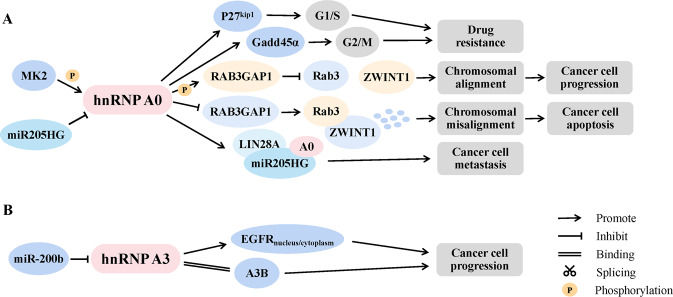
Mechanisms of hnRNPA0 and A3 in cancer based on available studies. **A** HnRNPA0 served as a strong promoter for various cancers. MK2-activated hnRNPA0 drove cancer cell drug resistance by regulating p27(Kip1) and Gadd45α. Meanwhile, phosphorylated hnRNPA0 promoted chromosomal alignment by hindering RAB3GAP1-mediated interaction between ZWINT-1 and Rab3. While, the oncogenic effects of hnRNPA0 could be repaired by lncRNA miR205HG. **B** HnRNPA3 could facilitate cancer progression by affecting EGFR subcellular localization and binding to A3B. In addition, hnRNPA3 was a negative target of EMT regulator miR-200b.

Actually, the study on hnRNPA0 in cancer is still in its infancy (Fig. 1A), and its specific mechanisms and molecular regulatory networks in the cancer process remain unclear, thus there is still a long way to go to reach the final clinical transformation.

### HnRNP A1

**Fig. 2:**
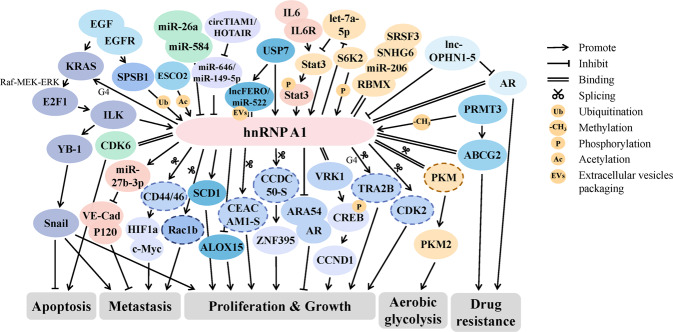
Simplified molecular regulatory networks of hnRNPA1 in cancer. The network of hnRNPA1 modulating cancer development are complex, regulating splicing, transcription, maturation, translation or EVs packaging. Additionally, the expression of hnRNPA1 could be influenced by certain upstream factors and post-translational modifications.

HnRNPA1 is one of the most abundant and ubiquitously expressed nuclear proteins. HnRNPA1-a (a short variant) and A1-b (a full-length variant) are the main variants that have been experimentally verified [4]. HnRNPA1 is the most well studied member of hnRNPA/B and plays a key role in a variety of cancers(Fig. 2). The ectopic expression of hnRNPA1 at different disease stages or sites has been progressively proved to be correlated with pathophysiological features and clinical prognosis of cancers, indicating hnRNPA1 as a promising cancer biomarker. Given its significance in cancer, hnRNPA1 has also been employed as a drug target in clinical trials, which may bring a new opportunity for cancer prevention and treatment in the near future.

### Expression, function and significance of hnRNPA1 as an oncogene in diverse cancers

As report goes, hnRNPA1 was the most frequently (76%) overexpressed hnRNPA/B family protein in non-small cell lung cancer (NSCLC) [32] and was negatively correlated with the overall survival of patients with lung cancer [33]. HnRNPA1 could augment the proliferation activity of lung cancer cells by directly binding to the 3ʹUTR of vaccinia related kinase 1 (VRK1) mRNA, expediting its translation and then increasing cyclin D1 expression [33]. Similarly, hnRNPA1 has also been found to be highly expressed in sentinel lymph nodes, tissues and serum of CRC patients [34, 35]. In promoting CRC course, hnRNPA1 could link to 3ʹUTR of autophagy-related gene 6 (ATG6) mRNA and mediate G4 formation of TRA2B [36]. These findings implied that hnRNPA1 may become a potential cancer biomarker and therapeutic target.

Furthermore, hnRNPA1 was reported to be bound and phosphorylated on novel Ser4/6 sites by fibroblast growth factor 2 (FGF-2)-induced S6 kinase 2 (S6K2) [37]. Meanwhile, the RNA binding activity of hnRNPA1 could be interfered with by protein arginine methyltransferase 3 (PRMT3) via methylation modification [38]. Moreover, the localization of hnRNPA1 and its impact on mRNA alternative splicing could be affected by establishment of cohesion 1 homolog 2 (ESCO2)-mediated acetylation [39] and SPRY domain-containing SOCS box protein 1 (SPSB1)-induced ubiquitylation [40].

Therefore, based on existing research, hnRNPA1 was identified as a novel cancer indicator. With the upstream mechanisms of hnRNPA1 being unveiled gradually, it is initially clear that multiple post-translational modifications are important factors affecting the stability and molecular function of hnRNPA1.

### The molecular mechanisms of hnRNPA1 involved in modulating cancer progression

Mechanically, hnRNPA1, a classical RBP, is involved in regulating the splicing and maturation of various key cancer genes, in which hnRNPA1 arginine methylation was found to play a prominent role [41]. In breast cancer (BC), hnRNPA1 affect the malignant properties of cancer cells by mediating the CEACAM1-S/-L ratio [42]. Likewise, the ratio of CCDC50-FL and CCDC50-S was also adjusted by hnRNPA1 in ccRCC, which could accelerate ccRCC progression through promoting the carcinogenic transformation of CCDC50-S [43]. Moreover, hnRNPA1 could interact with HPV18 exonic splicing silencer (ESS) [44] and HPV16 late regulatory element (LRE) [45], respectively, participating in balancing the splicing of HPV18 and HPV16 pre-mRNAs [46]. These ultimately catalyzed the malignant transformation of HPV and provided another potential target for HPV-related cancers. Additionally, the spliced variants of cyclin-dependent kinases 2 (CDK2) and transmembrane receptor for hyaluronic acid CD44 were both manipulated by hnRNPA1 to drive the development of oral squamous cell carcinoma (OSCC) and metastatic BC [47, 48].

It is worth noting that hnRNPA1 has been shown to alter aerobic glycolysis of cancer cells by directing the alternative splicing of pyruvate kinase (PKM) [49]. Concretely, the combination between the RGG motif of hnRNPA1 and the sequences flanking PKM exon 9 was enhanced by STAT3, thereby inhibiting PKM1 isoform formation and inducing PKM2 isoform production. However, this process was in turn blocked by microRNA let-7a-5p, thus forming a feedback loop between let-7a-5p, STAT3 and hnRNPA1 as a new way mediating aerobic glycolysis of BC [50]. As in other cancers, hnRNPA1-mediated variable splicing of PKM was essential for accelerating cellular glycolysis, and upstream promoters such as lncRNA SNHG6 and ESCO2, and repressors such as RBMX and miR-206 may affect the smooth advancement of this process by interacting with hnRNPA1 [39, 50–52].

Certainly, hnRNPA1 was also a critical mediator for multiple cancer regulators. For instance, hnRNPA1 could recognize the specific DNA conformation of KRAS, a G4 structure, and form an EGF-KRAS-ILK-hnRNPA1 regulatory loop to maintain the invasive activity of pancreatic ductal adenocarcinoma (PDAC) cells [53, 54]. Additionally, hnRNPA1 was involved in tumor immune responses as well. Ectopic hnRNPA1 elicited thapsigargin-induced endoplasmic reticulum (ER) stress, promoted translation of specific melanoma-overexpressed antigen 1 (MELOE-1), and further enhanced recognition of melanoma cells by MELOE-1-specific T-cell clone, improving their immune efficacy [55]. Moreover, hnRNPA1 played an important role in mediating hormone homeostasis. HnRNPA1 was found to selectively suppress androgen receptor (AR) transactivation via interruption of AR-ARA54 interaction and ARA54 homodimerization in prostate cancer (PCa) [56]. HnRNPA1 was also intimately involved in promoting intercellular communication between mesenchymal cancer cells and blood vessel endothelium. Detailedly, hnRNPA1 encapsulated miR-27b-3p into exosomes and delivered them into vascular endothelial cells, setting the stage for subsequent exosomal miR-27b-3p promotion of circulating tumor cell-mediated cancer cell metastasis [57]. Coincidentally, the effect of hnRNPA1 in assisting the packaging of different molecules into extracellular vesicles (EVs) was successively discovered. Currently, it was well documented that hnRNPA1 could be recruited by USP7 to facilitate exo-lncFERO and exo-miR-522 secretion, aiding their regulating of lipid metabolism, ferroptosis and individual chemosensitivity gastric cancer (GC) cells [58, 59]. Analogously, the role of hnRNPA1 loading batched miRNAs/lncRNAs into EVs was revealed in lung cancer, bladder cancer (BCa) and advanced head and neck cancer (HNC) as well [60–62]. More than that, hnRNPA1 was demonstrated to be concerned in mediating PCa enzalutamide (Enz) sensitivity via lnc-OPHN1-5/AR interaction [63], promoting triple-negative breast cancer (TNBC) progression via competitively binding to lncRNA HYOU1-AS [64], sustaining activation of NF-κB pathway in PDAC via lncRNA-PLACT1/IκBα/E2F1 feedback loop [65], and regulating ovarian cancer (OC) chemoresistance via miR-18a-KRAS axis [66].

Collectively, the molecular mechanisms of hnRNPA1 in cancer development are complicated, whether it is involved in pre-mRNA splicing and processing, competitively binding to varied RNAs, or assisting in EVs packaging and secretion. As the research on hnRNPA1 moves along, its application in cancers will attract more and more attention.

### Advances in drugs targeting hnRNPA1

**Fig. 3:**
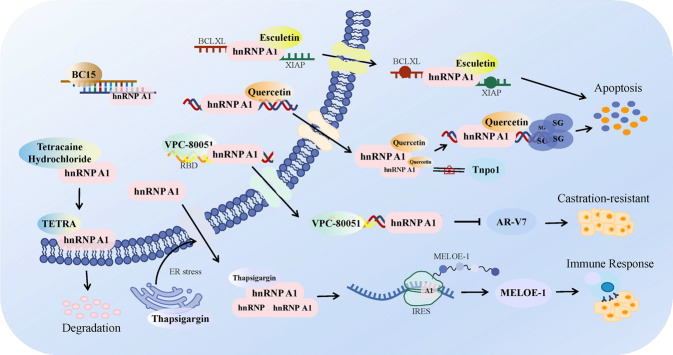
Advances in drugs targeting hnRNPA1. HnRNPA1 was identified as a great target for cancer therapy and a variety of hnRNPA1-targeting compounds were attempted to applied into clinic, including small molecule inhibitor VPC-80051, plant-extracted flavonoid quercetin, coumarin derivative esculetin, single-stranded DNA aptamer BC15 and even local anesthetic tetracaine.

In recognition of the importance of hnRNPA1, numerous hnRNPA1-targeting compounds have been developed successively for clinical treatment of cancers (Fig. 3). VPC-80051, the first small molecule inhibitor targeting hnRNPA1 RBD to be synthesized, could dramatically reduce androgen receptor AR-V7 messenger levels in castration-resistant prostate cancer (CRPC) cell lines and significantly improve the therapeutic effect of PCa [67]. Presently, many existing drugs, foods and plant ingredients have been unearthed in succession for cancer treatment by targeting hnRNPA1. In PCa, hnRNPA1 was identified as a direct anti-cancer target of quercetin, a flavonoid abundantly present in plants. Binding to the C-terminal region of hnRNPA1, quercetin hindered hnRNPA1’s combination with transportin 1 (Tnpo1), leading to its cytoplasmic retention and subsequent recruitment of hnRNPA1 to stress granules (SGs), ultimately putting cancer cells on the path to apoptosis [68]. Meanwhile, esculetin, a coumarin derivative from several herbs, was shown to induce apoptosis of endometrial cancer cells by affecting the nucleocytoplasmic transport of the hnRNPA1-BCLXL/XIAP mRNA complex [69]. Additionally, hnRNPA1-specific single-stranded DNA aptamer, BC15, was developed as a potential drug candidate for hepatocarcinoma treatment [70]. Of interest, tetracaine, a local anesthetic with potent anticancer effects, was reported to cause melanoma cell cycle arrest by driving hnRNPA1 accumulation at the nuclear envelope and weakening hnRNPA1 protein stability [71], providing new evidence for the potential benefits of applying local anesthetics in cancer patients.

With the deepening of clinical and basic research on hnRNPA1, its important role in cancer origination and progression continues to emerge. HnRNPA1 is a biomarker with considerable clinical transformation value, whether for cancer early screening or targeted therapy.

### HnRNPA2/B1

HnRNPA2/B1 and A1 are the two most studied members of the hnRNPA/B family. HnRNPA2/B1 gene generates four splice variants, namely A2, A2b, B1 and B1b [72]. Although there is only a 12 amino acid difference between hnRNPA2 and B1, their expression is not identical throughout the cell cycle, in different tissue types, and at different disease stages. It has been established that hnRNPA2 and B1 may have distinct functions due to their slightly different preferences for RNA sequences [73]. However, although some findings have highlighted the importance of considering the specific functions of hnRNPA2/B1 spliceoforms, most studies have not distinguished between these isoforms yet [74].

### HnRNPA2/B1 is recommended as a promising cancer biomarker

In manifold cancers, hnRNPA2/B1 has been shown to exhibit high expression level and to be strongly associated with clinic-pathological features and prognosis. During mammalian lung development, hnRNPA2/B1 presents a dynamic process, with increased level closely correlating to lung precancerous lesion and lung cancer progression [75, 76]. Meanwhile, the sensitivity of hnRNPA2/B1 in NSCLC was 84.8% in brushing and 80.8% in biopsies, while 66.7% and 75% in small cell lung cancer (SCLC), respectively [77]. Supported by extensive research data, hnRNPA2/B1 was considered an independent risk factor for lung cancer and could be applied for early assessment, disease surveillance and prognosis prediction of lung cancer [78, 79]. Analogously, hnRNPA2/B1 has been found to be elevated in both hepatitis virus-positive liver tissues and hepatocellular carcinoma (HCC) tissues. Interestingly, the localization of hnRNPA2/B1 was altered during the transition from hepatitis virus infection to poorly differentiated HCC, suggesting that hnRNPA2/B1 could be employed for assessing HCC risk [80]. Also, abnormal hnRNPA2/B1 was thought to serve as an oncogenic driver of glioblastoma and was correlated with poor prognosis [81]. HnRNPA2/B1 co-localization with c-myc, c-fos, p53, and Rb was translocated to the cytoplasm, through which hnRNPA2/B1 played a key role in the differentiation of GC cells [82].

In conclusion, growing numbers of basic and clinical data elucidate the potential and value of hnRNPA2/B1 as a biomarker of cancers, particularly lung cancer and HCC, emphasizing the feasibility of achieving the application of hnRNPA2/B1 in clinical practice.

### Molecular mechanisms of hnRNPA2/B1 as a cancer driver gene

Throughout previous studies, it is easy to find that hnRNPA2/B1 typically acts as a cancer driver gene and influences the biological behaviors of cancer cells mainly by modulating PI3K/Akt, Wnt/β-catenin, MAPK/ERK and other signaling cascades. For instance, in PDAC, cervical cancer and multiple myeloma (MM), hnRNPA2/B1 could promote cancer cell growth and metastasis and impair their sensitivity to gemcitabine, 5-fluorouracil (5-FU), oxaliplatin, lobaplatin and irinotecan by activating KRAS-PI3K interaction or regulating ILF3-mediated Akt signals [83–86]. HnRNPA2/B1 also could expedite cancer progression by controlling the ERK/snail, p53/HDM2 and Wnt/β-catenin signaling [87–89]. Moreover, the targets of hnRNPA2/B1 are rich and diverse. HnRNPA2/B1 could serve as a trigger for RNA switch to modulate the function of miRNAs or lncRNAs in cancer cells [90]. Illustratively, hnRNPA2/B1 affected the prognosis of ESCA by regulating the miR-17-92 cluster [91], facilitated the malignant phenotype of OC by activating Lin28B [92], and advanced lung cancer progression by contributing to miR-106b-5p maturation [88]. In some cases, the oncogenic roles and expression of hnRNPA2/B1 were instead impacted by certain upstream effectors. In triggering NSCLC growth, hnRNPA2/B1 could be acetylated by transcriptional co-activator p300 [93]. And in the process of hnRNPA2/B1 promoting VHLα translation in renal cancer, the hnRNPA2/B1 level was in turn repressed by elevated VHLα [94].

Absolutely, the initial role of hnRNPA2/B1 in alternative splicing is not negligible. HnRNPA2/B1 could specifically recognize the AUGGUA motif upstream of HPV-16 5ʹ-splice site SD3632 and inhibited HPV-16 L1 production, enabling HPV-16 to evade the immune system and establish long-term persistent infection [95]. Moreover, hnRNPA2/B1 could exclude cassette exon 11 from macrophage stimulating 1 receptor (MST1R) and resulted in the generation of recepteur d’origine nantais ∆165 (RON∆165) isoform [96]. Similarly, the exon selective splicing in the 5ʹUTR of TP53INP2 was a key event downstream of hnRNPA2 [97]. In addition, the oncogenic isoform 202 of the anti-apoptotic factor BIRC5 was also managed by hnRNPA2/B1 [98].

The two isoforms of hnRNPA2/B1, A2 and B1, were distinguished for exploration in some experiments. In an inflammation-induced mouse model, upregulated hnRNPA2 induced immortalized liver progenitor cell formation. This finding pointed out that it was hnRNPA1, but not B1, that reduced the dominant-negative isoform of A-Raf and led to activation of Raf-MEK-ERK pathway in GC [99]. Furthermore, the low level of hnRNPA2 was captured in paclitaxel-resistant OC cells and was considered to be an important hallmark of OC chemoresistance, in which the possible contribution of hnRNPB1 was not discussed [100].

However, hnRNPA2/B1 may even exert seemingly contradictory biological effects in the same cancer, particularly in BC. Most studies have shown that hnRNPA2/B1 was increased in BC [101], negatively correlated with cancer suppressor breast cancer susceptibility gene 1 (BRCA1) [102], and was a marker of poor prognosis in patients with BC [103]. Serving as a cancer promoter, hnRNPA2/B1 could force the autophagy, growth and endocrine resistance of BC cells [104–106]. In contrast, hnRNPA2/B1 was reported to be decreased in the Breast Cancer Integrative Platform and to have a dramatically inhibitory effect on the distant metastasis of TNBC [107]. Mechanically, hnRNPA2/B1 bound to BC cell metastasis booster profilin 2 (PFN2) directly and reduced its stability. Silencing hnRNPA2/B1 activated ERK-MAPK/Twist and GR-beta/TCF4 pathways, but inhibited STAT3 and WNT/TCF4 signaling pathways [107]. Therefore, the molecular mechanisms of hnRNPA2/B1 in BC are variable, and the final effect it produces may be the result of a dynamic balance, which demands more exploration.

Conclusively, hnRNPA2/B1 is an extremely prospective cancer driver. Its biological functions in cancers, especially in BC, are not unidirectional or unique. More systematic and in-depth studies are required in the future to provide more detailed theoretical support.

### The mechanisms of hnRNPA2/B1 as a “cooperator” in cancer progression

A wealth of data have indicated that hnRNPA2/B1 is involved in various cancer networks as a “cooperator”. In other words, hnRNPA2/B1 is a dominant mediator of diverse cancer driver genes. Such as, hnRNPA2/B1 was recruited by Nm23-H1 to co-regulate Sp1 translation and thus increased lung cancer cell malignant degree [108]. HnRNPA2/B1 was utilized by the ubiquitin-like protein interferon-stimulated gene 15 (ISG15) to enhance OC cell responses to cisplatin [109]. The antioxidant uncoupling protein 2 (UCP2) sustained the metabolic shift from mitochondrial oxidative phosphorylation (mtOXPHOS) to glycolysis in pancreatic cancer (PC) cells with the help of hnRNPA2/B1 [110], through which the Src family kinase Fyn could modulate PC cell apoptosis as well [111]. In addition, the formation of the MIR100HG/hnRNPA2B1/TCF7L2 forward-regulatory loop and the c-MYC/LINC01234/hnRNPA2B1/miR-106b-5p/Cry2/c-MYc positive-feedback loop could effectively accelerate disease progression in cancer patients [112]. In terms of cooperation with different types of RNAs, hnRNPA2/B1 could interact with Linc01232 [113], lncRNA H19 [114] and circMYH9 [115], and bolster their work in cancer evolvement. Interestingly, under the specific mediation of hnRNPA2/B1, H19 was observed could be wrapped into exosomes and promote gefitinib resistance in lung cancer [116].

HnRNPA2/B1 is a core component of animated RNA packaging and a key modulator of vesicular RNA sorting [117]. In addition to H19 described above, lncRNA LNMAT2 could also be loaded by hnRNPA2/B1 into BCa-secreted exosomes to expedite lymphangiogenesis and lymphatic metastasis [118]. Surely, not only on lncRNAs, but also hnRNPA2/B1 could act on miR-122-5p EXO-motif to induce hepatic metastasis of lung cancer [119], and similarly could motivate exo-miR-394-mediated M2 polarization of macrophages [120]. Moreover, tumor-derived EVs-miR-378a-3p was enriched by hnRNPA2/B1 overexpression as well [121].

**Fig. 4:**
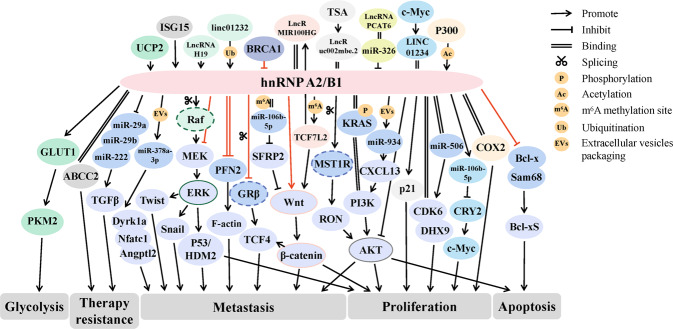
Representative molecular mechanisms of hnRNPA2/B1 in cancer. HnRNPA2/B1 affected the proliferation, metastasis, apoptosis, glycolysis and therapy resistance of cancer cells, mainly by regulating PI3K/Akt, Wnt/β-catenin, MAPK/ERK and other signaling pathways. HnRNPA2/B1 participated in cancer process not only as a cancer driver gene but also as a “cooperator” of other drivers such as ISG15, UCP2, MIR100HG, LINC01234, miR-934 and miR-378a-3p. Moreover, the stability and expression of hnRNPA2/B1 could be adjusted by Linc01232, P300, lncRNA H19 and so on. Among them, the function and mechanisms of hnRNPA2/B1 in BC remained controversial (highlighted with red).

The results above well illustrated the importance of hnRNPA2/B1 in the microscopic world of cancer molecular regulation. HnRNPA2/B1 is required in multiple aspects of cancer growth and development, the list goes on and on (Fig. 4).

### Therapeutic exploration targeting hnRNPA2/B1

**Fig. 5:**
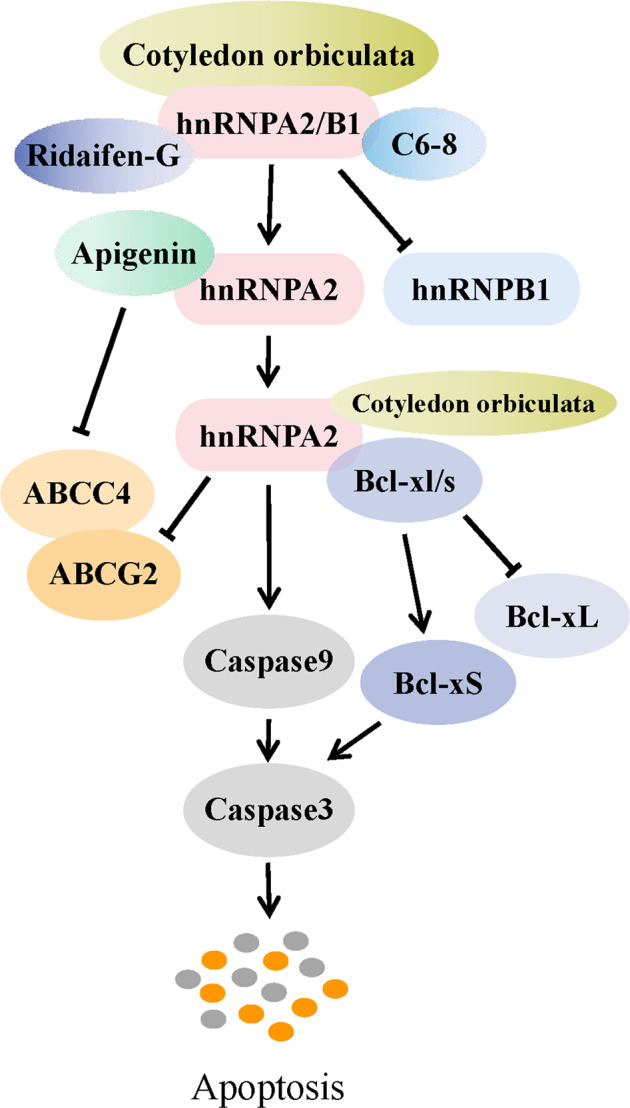
Therapeutic exploration targeting hnRNPA2/B1. The potential druggability of hnRNPA2/B1 was successively discovered. Cotyledon orbiculata could induce cancer cell apoptosis by splicing hnRNPA2/B1 from B1 to A2 isoform. Apigenin could influence chemosensitivity and apoptosis of cancer cells by regulating hnRNPA2. In addition, hnRNPA2/B1 was identified as a direct target of C6-8 and RID-G.

Considering the high impact of hnRNPA2/B1 on cancers, clinical attempts to target hnRNPA2/B1 are ongoing (Fig. 5). Cotyledon orbiculata, an extract of a South African medicinal plant, was revealed to induce apoptosis of CRC and ESCA cells by propelling hnRNPA2/B1 splicing from B1 to A2 [122]. Moreover, apigenin and other dietary flavones are emerging as potential chemo-sensitizers and have also been found to cause TNBC cell apoptosis by binding to hnRNPA2/B1 [123]. Specifically, hnRNPA2 deletion partially attenuated apigenin-induced sensitization of TNBC spheroids to doxorubicin through declining the efflux transporter ABCC4 and ABCG2. These findings provided a new perspective on the clinical value of hnRNPA2/B1 and underscored the rationality of using dietary compounds as chemotherapeutic adjuvants. In the course of investigating therapeutic strategies targeting hnRNPA2/B1, Li et al. [124]. identified that C6-8, an aptamer targeting ROS17/2.8 cells, could specifically bind to hnRNPA2/B1 and precisely label multiple cancer cell lines with fluorescent carbon nanodots (CDots) conjugation. In addition, hnRNPA2/B1 has also been discovered as a direct target candidate for tamoxifen analog Ridaifen-G (RID-G) in its potent anticancer working [125].

In general, hnRNPA2/B1 has shown the potential druggability for application as an excellent therapeutic target, meriting further investigations.

### HnRNP A3

HnRNPA3 is a relatively less studied member of the hnRNPA/B family and two isoforms, hnRNPA3a and 3b, have been reported, with hnRNPA3a being the only isoform detected in human cells [126]. HnRNPA3, with roughly the same structural features as other hnRNPA/B members, could bind to cis-acting response elements within mRNA 3ʹUTR and was important in the stable maintenance of telomere repeats and RNAs life cycle [127, 128]. For example, hnRNPA3 could recognize nuclear RNA export factor 7 (NXF7) in differentiated neuroblastoma cells and form a stable complex, taking part in the sorting, transport and/or storage of mRNAs [129].

Continuous research of hnRNPA3 has led to a growing understanding of its functions. In addition to its effects on cell senescence [130], differentiation [131] and neurodegeneration [132], hnRNPA3 is of great interest for its value in cancer (Fig. 1B). HnRNPA3 was found to increase gradually in the progression from cirrhosis, dysplastic nodules (DNs) and well-differentiated HCC to progressed HCC, and its expression level could be used to differentiate between high-grade dysplastic nodule (HGDN) and early HCC (eHCC), particularly in combination with glypican 3 (GPC3), with a specificity of 100%. Meanwhile, upregulated hnRNPA3 has been verified to strongly associate with poor survival of patients with HCC. Therefore, hnRNPA3 was proposed as a valuable differential diagnostic and prognostic biomarker during the multistep process of HCC carcinogenesis [133]. Furthermore, hnRNPA3 exhibited potential as a marker for advanced CRC in proteomics [134] and showed a high correlation with lymph node metastasis and poor prognosis in BCa patients undergoing radical cystectomy in a retrospective clinical study [135]. Simultaneously, hnRNPA3 was a key candidate protein related to BCa cisplatin resistance [136] and glioblastoma TMZ resistance [137], and was even a crucial regulator improving the efficacy of irinotecan enhanced by the traditional Chinese herbal preparation PHY906 [138]. These suggested that hnRNPA3 might have significant utility in clinical efficacy predicating. However, compared with that of other family members, the response of hnRNPA3 in lung cancer cell lines under acidosis, hypoxia, and serum deprivation conditions was the lowest and most constant [21], indicating that hnRNPA3 might be less sensitive in reflecting the survival status of cancer cells under stressful conditions.

Additionally, hnRNPA3 could significantly affect the subcellular localization of the classical oncogene EGFR. HnRNPA3 depletion reduced the nuclear accumulation of EGFR, accompanied by attenuated NSCLC growth vitality [139]. Also, hnRNPA3 was one of the downstream responders of miR-200b, a powerful regulator of the epithelial-mesenchymal transformation (EMT) in NSCLC [140]. Moreover, hnRNPA3 could also assist in the substantial elevation of APOBEC3B (A3B) in multiple cancers, which was a driver for the induction of unexpected mutation clusters [141].

In brief, research on hnRNPAs3 is still not in-depth, while hnRNPA3 shows great value in scientific studies and clinical applications. More efforts should be spent henceforth to comprehensively understand the specific function and molecular mechanisms of hnRNPA3.

### Interaction between members of the hnRNPA/B family

Although hnRNPA/B plays a key role in cancer progression, much remains to be discovered on how hnRNPA/B members interact with each other. Previously, some studies attempted to elucidate the interaction of hnRNPA/B. Among them, a protein interaction reporter (PIR)-based crosslinker was applied and thus hnRNPA1 and A2/B1 were shown to have a high level of amino acid sequence identity and both could crosslink with lysine residues K42 of hnRNPC [142]. Moreover, the Gly-rich domains of the two proteins were identified to bind to the trans-activation response DNA-binding protein 43 (TDP-43) [143] and H1-84mAb of influenza virus hemagglutinin [144], causing nervous system damage. According to the current researches, hnRNPA1 and A2/B1 were verified to co-localize with TDP-43 in the cytoplasm of atrophic muscle fibers [143], as well as with C9ORF72 [145], DNAJB6 [146] and SMN1 [147], respectively, in stress granules. Furthermore, hnRNPA1 and A2/B1 were frequently present in the same complex and cooperated in molecular biological functions, such as regulating the transcription of cancer suppressor ANXA7 [148], controlling the splicing response to oxaliplatin-mediated DNA damage [149], acting as inhibitors of HPV16 E7 expression [46], accelerating the transcriptional elongation of P-TEFb-dependent genes [150], participating in the reversal of 5-Fu resistance in cancer cells [151], and modulating alternative splicing of PKM2 in proliferating cells [152]. In addition, hnRNPA/B members A2/B1 and A3 were also found to be contained in the same complex [153] and involved in maintaining embryonic and adult cell stemness by interacting with SOX2 [154].

Notably, hnRNPA1 and A2/B1 were reported to regulate each other’s expression in a compensatory manner at both RNA and protein levels and were confirmed to be mediated by their respective 3ʹUTRs [155]. Moreover, there was a complementary relationship between hnRNPA1 and A2/B1. When hnRNPA1 was deficient, A2/B1 could compensate for the A1 deficiency to aid distal 5ʹ splice site selection [156].

Studies above have provided some evidence for synergistic interactions of hnRNPA/B family members. However, to the best of our knowledge, the specific underlying mechanisms of interaction between members of the hnRNPA/B family are still not explored, which may become a focus for future hnRNPA/B family-related studies.

## Summary and outlook

The paper presents a review on the relationship between the hnRNPA/B family and cancer occurrence and development, mainly focusing on the characteristic alterations and clinical significance of hnRNPA0, A1, A2/B1 and A3 in various cancers (Table 1) and comprehensively summarizing their biological functions and related molecular mechanisms involved (Table 2). HnRNPA/B can not only regulate the splicing, transcription, translation and translocation of targets but also coordinate or antagonize the roles of relevant functional genes in the malignant process of cancer.

**Table 1:** Expression characteristics and clinical significance of hnRNPA/B family in diverse cancers.

Cancer type	HnRNPs	Expression	Role	Clinical correlation	Detection methods	Ref.
MNs	hnRNPA0	Downregulated	Anti-cancer	Pathologic differentiation	Database	[23]
MM	hnRNPA2/B1	Upregulated	Oncogenic	OS	qRT-PCR	[86]
Glioblastoma	hnRNPA2/B1	Upregulated	Oncogenic	Survival	Database, qRT-PCR	[81]
OSCC	hnRNPA1	Upregulated	Oncogenic	Clinicopathologic stages	IHC, western blot	[47]
HNSCC	hnRNPA2/B1	Upregulated	-	OS, DSS	Database	[158]
LC	hnRNPA1	Upregulated	Oncogenic	OS	IHC, western blot, qRT-PCR	[32, 33]
	hnRNPA2/B1	Upregulated	Oncogenic	Lung development, early diagnosis, TNM stage, survival	Database, IHC, western blot; northern blot	[75, 77, 93, 159]
BC	hnRNPA2/B1	Conflicting	Conflicting	Survival	IHC, mRNA-sqe, northern blot	[101, 106, 107, 160]
ESCA	hnRNPA0	Upregulated	Oncogenic	-	western blot	[31]
	hnRNPA2/B1	Upregulated	Oncogenic	Disease risk, TMN stage; survival;	Database, IHC	[91, 161]
GC	hnRNPA1	Upregulated	Oncogenic	-	Database, IHC, western blot	[162]
	hnRNPA2/B1	Upregulated	Oncogenic	Pathologic differentiation, survival	Database, IHC, western blot, qRT-PCR, proteomics technique	[82, 98]
CRC	hnRNPA0	Upregulated	Oncogenic	Tumor size	Database, qRT-PCR	[28]
	hnRNPA1	Upregulated	Oncogenic	Lymph node metastasis, UICC staging, differentiation, recurrence, survival	IHC, western blot, qRT-PCR, proteomics technique	[34, 35, 163]
	hnRNPA2/B1	Upregulated	Oncogenic	Lymph node metastasis, distant metastasis	IHC, western blot	[164, 165]
	hnRNPA3	Upregulated	-	-	Proteomic technique	[134]
PC	hnRNPA2/B1	Upregulated	Oncogenic	Lymph node metastasis, pathologic differentiation	IHC	[166]
HCC	hnRNPA1	Upregulated	Oncogenic	-	Fluorescent probe	[70]
	hnRNPA2/B1	Upregulated	Oncogenic	Pathologic differentiation, hepatitis virus infection, survival	Database, IHC, western blot, qRT-PCR	[80, 99, 167]
	hnRNPA3	Upregulated	Oncogenic	Tumor size, differentiation, cirrhosis, diagnosis	IHC	[133]
PCa	hnRNPA1	Upregulated	Oncogenic	Gleason score, lymph node metastasis, advanced tumor stage, positive surgical margin, early biochemical recurrence	IHC	[168]
	hnRNPA2/B1	Upregulated	-	RFS	Database	[169]
OC	hnRNPA2/B1	Upregulated	Oncogenic	OS, PFS	Database	[92, 170]
ACC	hnRNPA2/B1	Upregulated	-	OS, EFS	Database	[171]
ccRCC	hnRNPA0	Upregulated	-	Pathologic differentiation, OS	Database	[26]
BCa	hnRNPA3	Upregulated	Oncogenic	Lymph node metastasis, PFS	IHC	[135]

*MNs* myeloid neoplasms, *MM* multiple myeloma, *OSCC* oral squamous cell carcinoma, *HNSCC* head and neck squamous cell carcinoma, *LC* lung cancer, *BC* breast cancer, *ESCA* esophageal carcinoma, *GC* gastric cancer, *CRC* colorectal cancer, *PC* pancreatic cancer, *HCC* hepatocellular carcinoma, *PCa* prostate cancer, *OC* ovarian cancer, *ACC* adrenocortical carcinoma, *ccRCC* clear cell renal cell carcinoma, *BCa* bladder cancer, *OS* overall survival, *DSS* disease-specific survival, *UICC* International Union against Cancer, *RFS* relapse-free survival, *PFS* progression-free survival, *EFS* event-free survival.

**Table 2:** Biological functions and related molecular mechanisms of hnRNPA/B involved in different cancers.

Cancer type	HnRNPs	Phenotypic effects	Related genes	Molecular function	Signal pathway	Ref.
MM	hnRNPA1	Glycolysis	NEK2, PKM	Binding mRNAs, regulating alternative splicing	-	[172]
	hnRNPA2/B1	Proliferation	ILF3, AKT3	Binding mRNAs and regulating targets	-	[86]
Glioblastoma	hnRNPA2/B1	Growth	c-FLIP, BIN1, WWOX, RON	Regulating alternative splicing	-	[81]
OSCC	hnRNPA1	Proliferation, cell cycle	CLASP1, NEK11, NEK9, NRAS, RCC2, SEPT3, SEPT8	Regulating G2/M related genes	-	[47]
HNC	hnRNPA1	Chemotherapy resistance	Exo-miR-196a	Exosome packaging	-	[62]
	hnRNPA2/B1	Metastasis	MST1R	Regulating alternative splicing	Akt/PKB signaling pathway	[96]
PTC	hnRNPA1	Proliferation, migration	miR-646	Serving as a direct target of miRNAs	-	[173]
LC	hnRNPA0	Cell cycle, chemotherapy resistance	MK2, p27, Gadd45α	Stabilizing mRNAs	-	[29, 30]
	hnRNPA1	Proliferation, invasion, migration	VRK1, EGF, SPSB1, Rac1, CD44, lncRNA SCIRT, miR-665, miR-149-5p	Binding mRNAs/miRNAs, regulating alternative splicing, modulating translation, exosome packaging	Ubiquitination pathway, EGF signaling pathway	[33, 40, 60,174–176]
	hnRNPA2/B1	Proliferation, invasion, migration, apoptosis, drugs resistance	E-cadherin, ERK, p53, HDM2, Nm23-H1, Sp1, COX-2, p300, miR-106b-5p, miR-506, CDK6, miR-122-5p, LINC01234, miR-106b-5p, lncRNA H19, lncRNA CACNA1G-AS1	Binding mRNAs/miRNAs/lncRNAs and regulating targets, mediating EMT, EVs packaging,	ERK, P53/HDM2, Akt and Wnt-β/catenin signaling pathway	[76, 87, 88, 90, 93, 108, 112, 116, 119, 177]
	hnRNPA3	Growth	EGFR, miR-200b	Modulating subcellular localization, serving as a target of miRNAs	-	[139, 140]
BC	hnRNPA1	Proliferation, invasion, glycolysis	CEACAM1, CD44, PKM2, miRNA let-7a-5p, Stat3, lncRNA HYOU1-AS	Binding and regulating targets, regulating alternative splicing,	-	[42, 48, 64, 178]
	hnRNPA2/B1	Proliferation, metastasis, therapy resistance	PFN2, lncRNA BC200, Bcl-x, STAT3, miR-29a-3p, miR-29b-3p, miR-222-3p, miR-1266-5p, miR-1268a, miR-671-3p, BRCA1	Binding mRNAs and regulating targets, mediating EMT, regulating alternative splicing	ERK-MAPK/Twist, GR-beta/TCF4, STAT3, WNT/TCF4, TGFβ and Akt signaling pathway	[102,104–107,160]
ESCA	hnRNPA0	Invasion, migration	lncRNA miR205HG, LIN28A	Binding lncRNAs, serving as a target of lncRNAs	-	[31]
	hnRNPA2/B1	Proliferation	miR-17-92	Regulating targets	-	[91]
GC	hnRNPA1	Proliferation, invasion, migration, chemotherapy resistance	lncFERO, SCD1, lncRNA SNHG8, miR-522, USP7, miR-339, miR-490, lncRNA CCAT1	Binding and regulating targets, mediating EMT, exosome packaging, serving as a target of miRNAs	-	[58, 59, 162,179–181]
	hnRNPA2/B1	Proliferation, metastasis, apoptosis, chemotherapy resistance	BIRC5	Regulating alternative splicing	-	[98]
CRC	hnRNPA0	Growth, cell cycle, apoptosis	RAB3GAP1, ZWINT1,	Promoting excessive mitosis, binding mRNAs	-	[28]
	hnRNPA1	Proliferation, metastasis, apoptosis, glycolysis	miR-18a, CDK6, TRA2B, miR-27b-3p, STAT3, miR-339-5p, PKM2, miR-206, lncRNA SNHG6, ATG6, S6K2	Binding mRNAs/miRNAs/lncRNAs, regulating alternative splicing, binding G4 structure, modulating transcription, exosome packaging, serving as a target of miRNAs	Autophagolysosomal degradation pathway, ERK/MAPK signaling pathway	[36, 51, 52, 57, 163, 165,182–185]
	hnRNPA2/B1	Growth, metastasis, chemotherapy resistance	lncRNA MIR100HG, TCF7L2, circMYH9, p53, lncRNA H19, Raf-1, miR-934, lncRNA RP11	Binding mRNA/lncRNAs and regulating targets, exosome packaging	Wnt/β-catenin, Raf/ERK and PI3K/Akt signaling pathway	[114, 115, 120, 164, 186]
PC	hnRNPA1	Invasion, migration, chemotherapy resistance	KRAS, PRMT3	Binding mRNAs, binding G4 structure,	-	[38, 54]
	hnRNPA2/B1	Proliferation, invasion, migration, apoptosis, chemotherapy resistance, glycolysis	KRAS, PI3K, E-cadherin, MMP-2, UCP2, GLUT1, PKM2, Linc01232, A-Raf, Fyn, Bcl-x	Binding mRNAs/lncRNAs and regulating targets, regulating alternative splicing, mediating EMT	PI3K/AKT/mTOR and A-Raf/ERK/MAPK/snail signaling pathway	[83, 84, 89, 110, 111, 113, 166]
HCC	hnRNPA1	Proliferation, migration	BC15	-	-	[70]
	hnRNPA2/B1	Proliferation, metastasis, apoptosis	miR-326, LncRNA-uc002mbe.2, Akt, p21, A-Raf	Binding lncRNAs, regulating alternative splicing, serving as a direct target of miRNAs	Raf-MEK-ERK signaling pathway	[99, 187, 188]
PCa	hnRNPA1	Growth, drug sensitivity	ARA54, lnc-OPHN1-5, AR	Binding mRNAs/lncRNAs and regulating targets	-	[56, 63]
	hnRNPA2/B1	Proliferation, metastasis	miR-378a-3p, CTNNB1	binding mRNA and regulating targets, EVs packaging	-	[121, 189]
OC	hnRNPA1	Chemotherapy resistance	miR-15a-5p, miR-25-3p, miR-18a-3p, KRAS	Binding miRNAs, serving as a direct target of miRNAs	-	[66]
	hnRNPA2/B1	Proliferation, metastasis, apoptosis, chemotherapy resistance	Lin28B, ISG15, ABCC2	Binding mRNAs and regulating targets, modulating translation	-	[92, 100, 109]
Cervical cancer	hnRNPA1	Proliferation, invasion, migration	EGF, SPSB1, Rac1, HPV18, HPV16, p300	Regulating alternative splicing, modulating translation	Ubiquitination pathway, EGF signaling pathway	[40, 44, 45, 190, 191]
	hnRNPA2/B1	Proliferation, cell cycle, invasion, apoptosis, chemotherapy sensitivity	PI3K, AKT, p21, p27	Targeting signaling pathway	PI3K/AKT signaling pathway	[85]
ccRCC	hnRNPA1	Proliferation, invasion, migration	CCDC50, ZNF395	Regulating alternative splicing	-	[43]
	hnRNPA2/B1	Proliferation, invasion, migration	VHLα, c-myc	Binding mRNAs and regulating targets, modulating translation	-	[94]
BCa	hnRNPA1	Proliferation, invasion, migration, glycolysis	RBMX, PKM, lncRNA BCYRN1, WNT5A, CD46, lncRNA ELNAT1,	Binding mRNAs, regulating alternative splicing, modulating translation, EVs packaging	Wnt/β-catenin signaling pathway	[50, 61, 192, 193]
	hnRNPA2/B1	Metastasis	lncRNA LNMAT2	Exosome packaging	-	[118]
Melanoma	hnRNPA1	-	MELOE-1	Binding mRNAs, modulating translation	-	[55]

*MM* Multiple myeloma, *OSCC* oral squamous cell carcinoma, *HNC* head and neck cancer, *PTC* papillary thyroid cancer, LC lung cancer*, BC* breast cancer*, ESCA* esophageal carcinoma*, GC* gastric cancer*, CRC* colorectal cancer, *PC* pancreatic cancer, *HCC* hepatocellular carcinoma*, PCa* prostate cancer*, OC* ovarian cancer, *ccRCC* clear cell renal cell carcinoma*, BCa* bladder cancer, *EMT* epithelial-mesenchymal transition, *EVs* extracellular vesicles.

Generally, hnRNPA/B exhibits a dynamic shift in human tissues. HnRNPA2/B1 was displaced and progressively elevated during the progression from precancerous lesions to advanced cancer stages, demonstrating its potential for dynamic cancer surveillance. Furthermore, hnRNPA3 combined with GPC3 can effectively differentiate between HGDN and eHCC, implying the aptitude of hnRNPA3 to differentially diagnose cancer. Whether other hnRNPA/B family members also present dynamic signature changes in other cancer progression remains to be further investigated.

HnRNPA/B is highly expressed in most cancers and is often predictive of disappointing survival and poor treatment outcomes. However, there are some contradictions. For instance, hnRNPA2/B1, generally upregulated in breast cancer, has been detected to be reduced in TNBC and negatively correlated with metastasis. Therefore, further distinguishing different pathological types, disease stages or treatment phases of cancer in future studies is necessary.

Furthermore, it is of fundamental significance to clarify the complex molecular mechanisms of hnRNPA/B. Firstly, in terms of the upstream mediums, the post-translational modifications cannot be ignored [157]. The level of hnRNPA/B can be manipulated as a result of ubiquitination, acetylation or phosphorylation. However, the available data are far from sufficient to explain the specific upstream mechanisms that shape hnRNPA/B in cancer. Secondly, hnRNPA/B participates in the entire process from RNA production to stabilization, and the molecular mechanisms involved are being discovered, but systematic and comprehensive research and summaries are not yet sufficient and more efforts are needed. In addition, the unknown network of interactions and mechanisms between members of the hnRNPA/B family is a novel topic worthy of further exploration in the future.

Having unraveling the potential of hnRNPA/B for clinical application, some investigators are beginning to devote themselves to exploring its targeted inhibitors or drugs. These include VPC-80051, BC15, quercetin, esculetin, kaempferol and tetracaine targeting hnRNPA1, and cotyledon orbiculata, C6-8, RID-G, apigenin and other dietary flavones targeting hnRNPA2/B1. These advances provide a starting point for conducting translational studies on hnRNPA/B.

In summary, breakthroughs in the comprehension of the role and mechanisms of hnRNPA/B in cancer malignant progression have yielded exceptional results in recent years. A large body of evidence suggests that hnRNPA/B, especially hnRNPA1 and A2/B1, have a good clinical value as a marker for early cancer diagnosis, disease monitoring, prognosis assessment and efficacy evaluation. It is very worthwhile to further explore hnRNPA/B, which will provide a new perspective for future individualized targeted cancer therapy, retaining very promising targets.

## Data Availability

The datasets used and/or analysed during the current study are available from the corresponding author on reasonable request.
